# Abnormal UP/DOWN Membrane Potential Dynamics Coupled with the Neocortical Slow Oscillation in Dentate Granule Cells during the Latent Phase of Temporal Lobe Epilepsy^1^,^2^,^3^

**DOI:** 10.1523/ENEURO.0017-16.2016

**Published:** 2016-05-31

**Authors:** David W. Ouedraogo, Pierre-Pascal Lenck-Santini, Geoffrey Marti, David Robbe, Valérie Crépel, Jérôme Epsztein

**Affiliations:** 1 Institut National de la Santé et de la Recherche Médicale U901, 13273 Marseille, France; 2 Unité Mixte de Recherche 901, Aix-Marseille University, 13273 Marseille, France; 3 Institut de neurobiologie de la méditerranée, 13273 Marseille, France; 4 Department of Neurological Sciences, University of Vermont, Burlington, Vermont 05405; 5 Centre National de la Recherche Scientifique, Institut des Sciences du Mouvement, Unité Mixte de Recherche 7287, Aix-Marseille University, 13288 Marseille, France

**Keywords:** dentate gyrus, epilepsy, in vivo whole-cell recordings, sleep, slow oscillation, UP/DOWN state

## Abstract

The dentate gyrus, a major entry point to the hippocampus, gates (or filters) incoming information from the cortex. During sleep or anesthesia, the slow-wave oscillation (SWO) orchestrates hippocampus–neocortex communication, which is important for memory formation. The dentate gate is altered in temporal lobe epilepsy (TLE) early during epileptogenesis, which favors the propagation of pathological activities. Yet, whether the gating of physiological SWO by dentate granule cells (DGCs) is altered in TLE has remained unexplored. We combined intracellular recordings of membrane potential (*V*_m_) of DGCs and local field potential recordings of the SWO in parietal cortex in anesthetized rats early during epileptogenesis [post-status epilepticus (SE) rats]. As expected, in control rats, the *V*_m_ of DGCs weakly and rarely oscillated in the SWO frequency range. In contrast, in post-SE rats, the *V*_m_ of DGCs displayed strong and long-lasting SWO. In these cells, clear UP and DOWN states, in phase with the neocortical SWO, led to a bimodal *V*_m_ distribution. In post-SE rats, the firing of DGCs was increased and more temporally modulated by the neocortical SWO. We conclude that UP/DOWN state dynamics dominate the *V*_m_ of DGCs and firing early during epileptogenesis. This abnormally strong neocortical influence on the dynamics of DGCs may profoundly modify the hippocampus–neocortex dialogue during sleep and associated cognitive functions.

## Abstract (precis)

The dentate gyrus, a major entry point to the hippocampus, gates (or filters) incoming information from the cortex. During sleep or anesthesia, the slow-wave oscillation (SWO) orchestrates hippocampus–neocortex communication, which is important for memory formation.

## Visual Abstract

**Figure:**
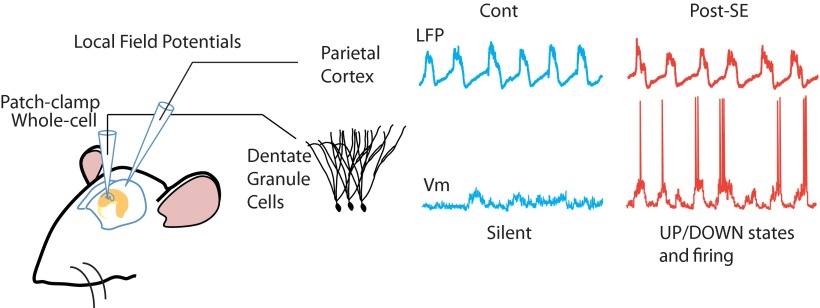

## Significance Statement

Communication between cortex and hippocampus during sleep, orchestrated by the neocortical slow-wave oscillation (SWO), is important for memory consolidation. Whether this communication is affected in temporal lobe epilepsy (TLE), a disease with profound memory impairments, is not known. In control rats, dentate granule cells (DGCs), at the gate of the hippocampus, filter incoming information from the cortex. This relative independence of hippocampal neurons from SWO allows the replay of hippocampus-specific information independently from the neocortex. Here, using *in vivo* whole-cell patch-clamp recordings of DGCs and field recordings in the neocortex, we report an abnormally strong influence of neocortical SWO on the membrane potential and firing of DGCs in TLE rats. This could profoundly alter hippocampus–neocortex dialogue during sleep and associated cognitive functions.

## Introduction

During slow-wave sleep and anesthesia, neocortical networks are dominated by the slow oscillation [slow-wave oscillation (SWO), 0.1-2 Hz; Steriade et al., 1993, 2001; Cowan and Wilson, 1994]. At the cellular level, the membrane potential (*V*_m_) of nearly all neocortical neurons synchronously alternates between epochs of steady depolarization (the UP state), associated with firing, and epochs of long-lasting hyperpolarization and quiescence (the DOWN state), leading to a bimodal distribution of their *V*_m_ (Timofeev et al., 2001; Volgushev et al., 2006; Rudolph et al., 2007; Chauvette et al., 2011). The SWO is not confined to neocortical networks and propagates to other subcortical (Wilson and Kawaguchi, 1996; Collins et al., 2001; Loewenstein et al., 2005; Mahon et al., 2006; Ros et al., 2009) and paleocortical (Isomura et al., 2006; Hahn et al., 2012) structures, where the *V*_m_ of principal cells also rhythmically alternates between UP and DOWN states. The SWO also propagates to the hippocampal formation (Isomura et al., 2006; Wolansky et al., 2006; Hahn et al., 2007). However, in contrast to cells in neocortical, subcortical, and paleocortical areas, the *V*_m_ of hippocampal principal neurons does not display rhythmic alternations between UP and DOWN phases for prolonged periods of time and lacks bimodality (unlike hippocampal interneurons, see Hahn et al., 2006). This suggests a lower impact of neocortical SWO on the *V*_m_ dynamics of hippocampal principal neurons than in other neocortical and subcortical structures. In line with this, the hippocampus can sometimes escape from the neocortical influence and generate specific activity patterns such as sharp-wave ripples during the neocortical silent (DOWN) state (Isomura et al., 2006).

The aforementioned relative uncoupling of the hippocampus with neocortical SWO is in agreement with the gating function of the dentate gyrus, a major entry point to the hippocampus (Acsády and Kali, 2007). Indeed, dentate granule cells (DGCs) are relatively silent [e.g., firing no or few action potentials (APs) spontaneously] in awake, anesthetized, and sleeping animals (Jung and McNaughton, 1993; Leutgeb et al., 2007; Neunuebel and Knierim, 2012; Pernía-Andrade and Jonas, 2014; Diamantaki et al., 2016; Kowalski et al., 2016) and the proportion of active cells in a given environment is low (Chawla et al., 2005). In temporal lobe epilepsy (TLE), the dentate gyrus undergoes profound structural and functional network modifications (Dudek and Sutula, 2007). Several subtypes of inhibitory interneurons are lost (Sloviter, 1987; Houser and Esclapez, 1996), and the axons of DGCs (the mossy fibers) sprout to form a direct excitatory feedback circuit not present in controls (Tauck and Nadler, 1985; Represa et al., 1987; Scharfman et al., 2003; Epsztein et al., 2005). Altogether, these morphofunctional changes increase the excitability of DGCs (Artinian et al., 2011) and render the dentate “gate” more permissive to the propagation of neocortical pathological activities, such as epileptiform discharges (Nagao et al., 1996; Behr et al., 1998; Pathak et al., 2007; Bragin et al., 2012; Krook-Magnuson et al., 2015). These changes and associated gating abnormalities start to be observed very early during epileptogenesis (Wuarin and Dudek, 2001; Kobayashi and Buckmaster, 2003; Kobayashi et al., 2003; Pathak et al., 2007), and notably before the recording of the first spontaneous seizures (the latent period). Spatial memory deficits associated with the disease can already be observed at this early stage (Chauvière et al., 2009). Here we asked whether the impact of the physiological SWO on the dynamics of the *V*_m_ and firing of DGCs could be altered early during epileptogenesis.

To address this question, we combined whole-cell patch-clamp recordings of DGCs *in vivo* together with neocortical local field potentials (LFPs) recordings of the SWO in control and TLE rats under anesthesia during the latent phase of TLE [post-status epilepticus (SE) rats]. In contrast to control rats, the *V*_m_ of DGCs in post-SE rats showed strong UP/DOWN state dynamics that were time locked to the neocortical SWO. Furthermore, the firing of DGCs was increased and more strongly paced by the SWO in post-SE rats. We conclude that the processing of SWO, an important physiological pattern of neocortical activity during sleep, by DGCs is impaired early during epileptogenesis.

## Materials and Methods

### Animals

All experiments were approved by the Institut National de la Santé et de la Recherche Médicale (INSERM) animal care and use committee and in accordance with the European community council directives (2010/63/UE). Data were obtained from male Wistar rats between the ages of postnatal day 27 (P27) to P43 (weight range, 100–130 g). We used the lithium/pilocarpine model of temporal lobe epilepsy with a ramp induction protocol. Rats were first injected with lithium chloride (3 mEq/kg). Fourteen to eighteen hours later, they were pretreated with methylscopolamine nitrate (1 mg/kg) to reduce the peripheral effects of pilocarpine. Thirty minutes after, they received a first low dose of pilocarpine (10 mg/kg). The same pilocarpine dose was repeatedly injected every 30 min until the behavioral manifestation of the first seizure was observed. The first seizure usually appeared after three to four doses of pilocarpine (30-40 mg/kg). This allowed us to take into account the individual differences in pilocarpine susceptibility between rats. One hour after the onset of status epilepticus, diazepam (8 mg/kg) was administered to stop the seizure. Recordings were performed 6-12 d after the status epilepticus. During this period, only interictal-like activities (ILAs), not seizures, could be recorded (mean frequency, 0.15 ± 0.07 Hz; ∼9 ILAs/min; *n* = 19 rats).

### Surgery

Animals were anesthetized with 1.5-2 g/kg urethane. The level of anesthesia was assessed using tail or foot pinching, body temperature, and recordings of the neocortical local field potentials. Body temperature was maintained at 37°C with a heating blanket (FHC). The animals were head fixed in a stereotaxic apparatus (SR-6, Narashige). A local analgesic (lidocaine) was applied as a gel on the ear bars to reduce pain during head fixation and was injected as a liquid below the skin before the first incision. An ophthalmic gel was applied to the eyes to prevent them from drying out during the surgery, and the eyes were covered with a piece of cardboard to protect them from the surgical light. The skull was exposed, and two small craniotomies (1.5 mm diameter) were drilled above the right hippocampus (−3.5 mm posterior to bregma; 2.5 mm lateral to bregma) to record in the dentate gyrus (∼3 mm deep from brain surface) and above the parietal cortex (−4 mm posterior to bregma; 4 mm lateral to bregma) to record in the parietal cortex (1 mm deep from brain surface with an anteroposterior angle of 20°).

### *In vivo* patch-clamp recordings

The *V*_m_ of DGCs was recorded in current-clamp mode using standard techniques for blind *in vivo* whole-cell recordings (Margrie et al., 2002). Borosilicate glass patch pipettes (resistance, 7–10 MΩ) were filled with a solution containing the following (in mm): 130 KMeSO4, 5 KCl, 5 NaCl, 10 HEPES-K, 2.5 MgATP, 0.3 NaGTP, 0.2 EGTA, and 0.1% biocytin, pH 7.25, adjusted with KOH. The *V*_m_ was amplified by an Axoclamp-2B Amplifier (Molecular Devices), low-pass filtered at 3 kHz, and digitized with a Digidata 1440A Digitizer (Molecular Devices) at 20 kHz. Only cells with overshooting APs, resting *V*_m_ less than −55 mV, and series resistance (R_s_) <100 MΩ were kept for further analysis. The *V*_m_ was corrected for a 10 mV liquid junction potential.

### LFPs and MUA recordings

LFPs and multiunit activity (MUA) were recorded from the parietal cortex and dentate granule cell layer using a glass electrode (∼5 MΩ) filled with Ringer’s solution (135 mm NaCl, 5.4 mm KCl, 1 mm MgCl_2_, 1.8 mm CaCl_2_, and 5 mm HEPES, pH adjusted to 7.2 with NaOH, and target osmolarity of 290 mmol/kg). LFPs and MUA were amplified 1000 times using a DAM80 amplifier (World Precision Instruments), bandpass filtered (0.1-3 kHz), digitized with a Digidata 1440A Digitizer (Molecular Devices), and sampled at 20 kHz. At the end of the recording, the pipette was submerged in a solution containing the red fluorescent dye 1,1'-dioctadecyl-3,3,3',3'-tetramethylindocarbocyanine perchlorate (DiI; Invitrogen), while the rat was still in the stereotaxic frame and reinserted at the recording depth for subsequent histological localization. Electrophysiological signals before and after DiI staining were similar.

### Morphology of the recorded cells

At the end of the recordings, animals were injected with an overdose of urethane and transcardially perfused with PBS 1× solution followed by 4% paraformaldehyde. The next day, 100-µm-thick coronal slices were cut and processed with the avidin-biotin-CY3 method to visualize biocytin-filled neurons *post hoc*. The morphology of the recorded cells was reconstructed using Neurolucida software (MBF Bioscience).

### Data analysis

Analyses were performed using custom-written programs in MATLAB (MathWorks), Origin (OriginLab), or Clampfit 10.4 (Molecular Devices).

#### Intrinsic intracellular properties

R_S_ values were not compensated for during the recording, but were calculated and compensated for off-line (Crochet and Petersen, 2006). To calculate R_S_ and membrane input resistance (R_N_) values, we used voltage responses to multiple hyperpolarizing and depolarizing current steps (100 pA) injected shortly after breaking in. The voltage values used to calculate R_S_ (V_S_) were found by exponentially fitting the voltage response (avoiding the first 2 ms during which the time constants resulting from pipette access resistance will dominate) to each current step. The exponential curve was then back-extrapolated to the start time of the step. The intersection of the extrapolated curve with the current step onset time gave V_S_ for each current step, which corresponds to the voltage drop across the R_S_. R_S_ was then calculated as the slope of the linear fit of the *I–*V_S_ curve and used to correct *V*_m_ off-line by subtraction whenever current was injected through the patch pipette. After R_S_ off-line compensation, the R_N_ was calculated for each cell. The voltage values used to determine R_N_ (*V*_N_) were calculated for each step as the difference in *V*_m_ between the baseline (before the current pulse) and steady-state voltage (100 ms after the start of the current pulse). The R_N_ was also calculated not on a single step but by using all *V*_N_ values, as the slope of the corresponding *I–V*_N_ curve. The membrane time constant was given by the time constant of the exponential fit of the *V*_N_ response to a 100 pA hyperpolarizing pulse. No holding current was injected during the recordings except when otherwise indicated. Resting membrane potential values were calculated as the mean of the Gaussian fit of *V*_m_ distribution using >30-s-long epochs. If the *V*_m_ distribution was best fitted by two Gaussian curves (bimodal cells), we used the voltage corresponding to the peak of the Gaussian curve fitting the lowest *V*_m_ hump values (the DOWN state). For all *V*_m_ analysis, the signal was detrended to remove ultraslow (<0.1 Hz) baseline fluctuations by low-pass filtering (0.1 Hz) then subtracting the low-pass-filtered data to the raw data. Threshold values were calculated using the first action potential in response to depolarizing steps of current as the voltage value at which the rate of depolarization (dV/dt) crossed 10 V/s.

#### Phase histograms

The times of intracellular APs were detected in high-pass-filtered (300 Hz) *V*_m_ as events higher than a predefined threshold. The times of MUA were determined in high-pass-filtered (1000 Hz) LFPs as events with amplitudes higher than five times the SD of the noise. The level of noise (and thus the detection threshold) was not significantly different between control and post-SE conditions (*p* = 0.81; *n* = 6 control rats, *n* = 6 post-SE rats). The phase of APs and MUA relative to the SWO cycle was derived from a Hilbert transformation of the LFP using the signal-processing toolbox in MATLAB. The peaks of the UP states are defined as 0° after the Hilbert transformation, and the troughs of the DOWN states are 180°. Phase modulation was evaluated by applying Rayleigh circular statistics (Fisher, 1993) .

#### Slow oscillatory epoch detection

**Figure 1.**
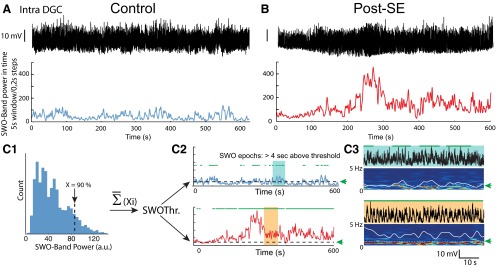
Method to compare the incidence and power of slow oscillations in the membrane potential of dentate granule cells from control and post-SE rats. ***A***, Top, *V*_m_ of a DGC from a control rat. The recording was bandpass filtered (0.1-40 Hz). Bottom, Time-varying power in the slow-frequency range (0.1-2 Hz) extracted from the time–frequency spectrogram of the *V*_m_ trace computed over a 5 s sliding window in 0.2 s steps. ***B***, Same as in ***A*** except that the cell from a post-SE rat was spontaneously firing (unlike the cell in ***A***), and spikes were digitally removed from the recording. Scale bar, as in ***A***. ***C1***, For each recording in controls, the 90th percentile highest value of the SWO band (0.1-2 Hz) power (*Xi*) was determined. The mean of all these values for control cells gives a unique threshold (SWOThr), which is then used to determine the SWO epochs in all DGCs from both control and post-SE rats. ***C2***, Example of the time-varying SWO power for the two recordings shown in ***A*** (top, blue) and ***B*** (bottom, red). Dashed lines correspond to the threshold for significant SWO epochs detection (green arrow). The epochs of high SWO power (green horizontal bars) are defined as epochs when the SWO power is above the threshold for >4 s. ***C3***, Illustration of detected SWO epochs corresponding to the light blue- and orange-shaded areas in ***C2***, Top, *V*_m_ traces. Bottom, Time–frequency spectrogram. Superimposed white lines represent the time-varying power values in the SWO band (0.1-2 Hz). Dashed white lines (green arrows) indicate the threshold used for SWO epochs detection (horizontal green bars above the traces).

Epochs of significant slow oscillations were detected using temporal spectral analysis of *V*_m_ in the SWO band (0.1-2 Hz; Fig. 1). Briefly, APs and hyperpolarizing steps used to probe the R_S_ during the recording were removed by linearly interpolating the signal. The time–frequency power spectrogram of *V*_m_ was computed using a 5 s sliding window advancing in steps of 0.2 s, and the time-varying power in the SWO frequency band (0.1-2 Hz) was extracted (Fig. 1*A*,*B*, bottom). For each control DGC, the value (*Xi*) corresponding to the highest 90% of all power values in the SWO frequency band was determined (Fig. 1*C1*). The average of all *Xi* values from control DGCs was then used as a unique threshold for SWO epoch detection in control and post-SE DGCs. The detection was performed on the time-varying power in the SWO frequency band (Fig. 1*C2*; see also Sales-Carbonell et al., 2013). A candidate SWO epoch should be above threshold for at least 4 s (e.g., approximately three oscillation cycles) to be considered as significant. For DGCs that spontaneously fired in post-SE rats, the results were not significantly different if APs were not artificially removed from the data (power in SWO band: Student’s *t* test, *p* = 0.92^mm^; duration of SWO epochs: Student’s *t* test, *p* = 0.89^nn^; percentage of SWO: Mann–Whitney rank sum test, *p* = 0.96^oo^; *n* = 8).

#### V_m_ distribution

To determine whether the *V*_m_ of a DGC was unimodally distributed, the Hartigan dip test (Hartigan and Hartigan, 1985) was performed on the distribution of a >30 s low-pass-filtered (40 Hz) *V*_m_ epoch centered on epochs of significant slow oscillatory content using the Matlab routine by Nicholas Price (Monash University; http://www.nicprice.net/diptest/). Nonunimodal cells were classified as bimodal if their *V*_m_ distribution was best fitted by the sum of two Gaussian curves. Unimodal cells were further qualified as skewed if their skewness, measured with the “skewness” function of the MATLAB statistics toolbox, was >0.5.

#### LFP UP–DOWN state detection

UP and DOWN states were detected using the bimodal distribution of neocortical LFP values as previously described (Volgushev et al., 2006). Briefly, the level for DOWN state detection was set at the lower two-thirds of the distance between the peaks of the bimodal distribution of LFP values. The level for UP state detection was set at the higher two-thirds of the distance between the peaks of the bimodal distribution of LFP values (see Fig. 4*A*,*B*). States were defined as epochs above the threshold for UP states or below the threshold for DOWN states lasting >200 ms and were terminated when the threshold was crossed for >40 ms.

#### DOWN–UP transitions triggered V_m_ averages

The amplitude of *V*_m_ varies with time and across different cells. To compute the DOWN–UP transition (DUT)-triggered *V*_m_ averages, APs were removed from the signal by linear interpolation and subthreshold *V*_m_ values were converted into dimensionless units of *z*-score as follows: *Z*(*x*) *=* (*x* − mean(*x*))/SD(*x*), where *x* is the *V*_m_, mean is the average value of the variable, and SD is the standard deviation. Thus, the amplitude of *V*_m_ is represented in the units of *z*, thereby allowing easy comparison across different cells. The times of neocortical DOWN state endings were used as transition points from the DOWN to the UP state. The values of z-scored *V*_m_, centered on the DOWN–UP transitions of neocortical SWO, were averaged across all detected neocortical DOWN–UP transitions.

#### Statistics

Statistical analyses were performed using SigmaStat (Systat Software), Statistica (Statsoft), and Prism (GraphPad) softwares (Table 1). For comparisons between groups with normal distribution and equal variance, the two-sample unpaired Student’s *t* test was used. When data were not normally distributed or different variances were calculated between groups, the Mann–Whitney rank sum test was used. For comparisons before and after depolarization, the paired Student’s *t* test was used when values were normally distributed; otherwise, the Wilcoxon signed rank test was used. The level of significance was set at *p* < 0.05. If not stated otherwise, *n* refers to the number of cells. The power of statistical tests used was calculated using GraphPad StateMat software (GraphPad). Values are given as the mean ± SEM.

**Table 1:** Statistical table

	Data structure	Type of test	Power or 25-75% confidence intervals
*a*	Normality test: passed (*p* = 0.12)Equal variance test: failed (*p* < 0.05)	Mann–Whitney rank sum test	25-75% control: 7–10.5; post-SE: 12.5–64.6
*b*	Normality test: passed (*p* = 0.16)Equal variance test: passed (*p* = 0.36)	Two-sample Student’s *t* test	0.99
*c*	Normality test: passed (*p* = 0.61)Equal variance test: failed (*p* < 0.05)	Mann–Whitney rank sum test	25-75% control: 7–45.3; post-SE: 54.6–109
*d*	Normality test: passed (*p* = 0,18)Equal variance test: passed (*p* = 0.44)	Two-sample Student’s *t* test	0.88
*e*	Normality test: passed (*p* = 0.36)Equal variance test: failed (*p* < 0.05)	Mann–Whitney rank sum test	25-75% control: −0.14 to −0.08; post-SE: −0.36 to −0.15
*f*	Normality test: passed (*p* = 0.59)Equal variance test: passed (*p* = 0.80)	Two-sample Student’s *t* test	0.30
*g*	Normality test: passed (*p* = 0.32)Equal variance test: failed (*p* < 0.05)	Mann–Whitney rank sum test	25-75% control: −43.5 to −39.0; post-SE: −39.9 to −37.4
*h*	Normality test: passed (*p* = 0.056)Equal variance test: passed (*p* = 0.82)	Two-sample Student’s *t* test	0.20
*i*	Normality test: passed (*p* = 0.37)Equal variance test: passed (*p* = 0.73)	Two-sample Student’s *t* test	0.20
*j*	Normality test: passed (*p* = 0.49)Equal variance test: passed (*p* = 0.36)	Two-sample Student’s *t* test	0.60
*k*	Normality test: failed (*p* < 0.05)	Mann–Whitney rank sum test	25-75% control: 7.75–10.4; post-SE: 9.4–16.8
*l*	Normality test: passed (*p* = 0.13)Equal variance test: passed (*p* = 0.26)	Two-sample Student’s *t* test	0.99
*m*	Normality test: passed (*p* = 0.44)Equal variance test: passed (*p* = 0.18)	Two-sample Student’s *t* test	0.70
*n*	Normality test: passed (*p* = 0.054)Equal variance test: passed (*p* = 0.22)	Two-sample Student’s *t* test	0.52
*o*	Normality test: passed (*p* = 0.10)Equal variance test: passed (*p* = 0.56)	Two-sample Student’s *t* test	0.30
*p*	Normality test: passed (*p* = 0.25)Equal variance test: passed (*p* = 0.52)	Two-sample Student’s *t* test	0.90
*q*	Normality test: passed (*p* = 0.12)	One-sample Student’s *t* test	0.99
*r*	Normality test: failed (*p* = 0.008)	One-sample Wilcoxon signed rank test	25–75%: post-SE: 0.37–0.55
*s*	Normality test: passed (*p* = 0.85)Equal variance test: passed (*p* = 0.44)	Two-sample Student’s *t* test	0.99
*t*	Normality test: failed (*p* < 0.05)	Mann–Whitney rank sum test	25–75% control: 0–0; post-SE: 0.02–0.76
*u*	Normality test: failed (*p* < 0.05)	Mann–Whitney rank sum test	25–75% control: 0–0; post-SE: 0.32–17.9
*v*	Normality test: failed (*p* < 0.05)	Mann–Whitney rank sum test	25–75% control: 1.15–1.27; post-SE: 1.17–1.33
*w*	Normality test: failed (*p* < 0.05)	Mann–Whitney rank sum test	25–75% control: 0–0; control depolarization: 0.16–0.43
*x*	Normality test: failed (*p* < 0.05)	Mann–Whitney rank sum test	25–75% post-SE: 0.02–0.76; control depolarization: 0.16–0.43
*y*	Normality test: passed (*p* = 0.099)	Paired *t* test	0.94
*z*	Normality test: passed (*p* = 0.645)Equal variance test: passed (*p* = 0.709)	Two-sample Student’s *t* test	0.99
*aa*	Normality test: passed (*p* = 0.64)Equal variance test: passed (*p* = 0.602)	Two-sample Student’s *t* test	0.99
*bb*	Normality test: passed (*p* = 0.48)Equal variance test: failed (*p* < 0.05)	Mann–Whitney rank sum test	25–75% control: 0.68–2.62; post-SE: 2.30–13.1
*cc*	Normality test: passed (*p* = 0.73)Equal variance test: failed (*p* < 0.05)	Mann–Whitney rank sum test	25–75% control: 0.21–0.53; post-SE: 0.15–0.23
*dd*	Normality test: failed (*p* < 0.05)	Mann–Whitney rank sum test	25–75% control: 97.8–193; post-SE: 66.7–229
*ee*	Normality test: passed (*p* = 0.61)Equal variance test: failed (*p* < 0.05)	Mann–Whitney rank sum test	25–75% control: 71.1–74.8; post-SE: 55.6–69.0
*ff*	Normality test: failed (*p* < 0.05)	Mann–Whitney rank sum test	25–75% control: 3.27–8.72; post-SE: 3.9–8.2
*gg*	Normality test: passed (*p* = 0.43)Equal variance test: passed (*p* = 0.25)	Two-sample Student’s *t* test	0.99
*hh*	Normality test: passed (*p* = 0.79)Equal variance test: passed (*p* = 0.24)	Two-sample Student’s *t* test	0.95
*ii*	Normality test: passed (*p* = 0.70)Equal variance test: passed (*p* = 0.42)	Two-sample Student’s *t* test	0.99

*jj*	Normality test: passed (*p* = 0.23)	Paired *t* test	0.99
*kk*	Normality test: failed (*p* < 0.05)	Wilcoxon signed rank test	25–75% control: 7.04–10.46; control depolarization: 7.62–14.4
*ll*	Normality test: passed (*p* = 0.56)	Paired *t* test	0.98
*mm*	Normality test: passed (*p* = 0.22)Equal variance test: passed (*p* = 0.92)	Two-sample Student’s *t* test	0.80
*nn*	Normality test: passed (*p* = 0.14)Equal variance test: passed (*p* = 0.83)	Two-sample Student’s *t* test	0.40
*oo*	Normality test: failed (*p* < 0.05)	Mann–Whitney rank sum test	25–75% post-SE no spike: 48.5–82.9;post-SE with spikes: 48.2–82.7
*pp*	Normality test: failed (*p* < 0.05)	Mann–Whitney rank sum test	25–75% post-SE: 0.32–17.9; control depolarization: 7.61–12.9

### Pharmacological agents

All drugs (lithium chloride, scopolamine, pilocarpine, diazepam, and urethane) were diluted in NaCl 0.9% and administered intraperitoneally. Scopolamine methyl nitrate and pilocarpine hydrochloride were purchased from Sigma-Aldrich; diazepam was purchased from Roche; streptavidin conjugated to Cy3 was purchased from Jackson ImmunoResearch; and DiI was purchased from Life Technologies.

## Results

### UP and DOWN state dynamics of dentate granule cell membrane potential in post-SE rats

**Figure 2.**
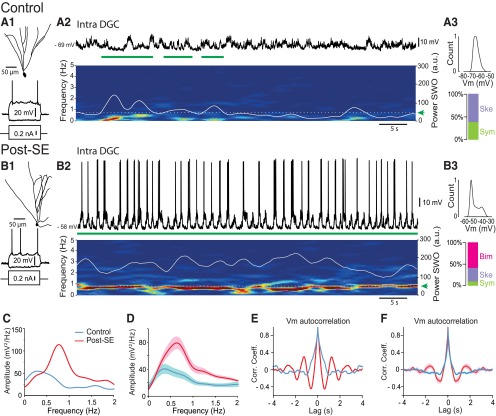
Increased slow-oscillatory UP/DOWN state dynamics of the membrane potential of dentate granule cells in post-SE rats. ***A1***, Neurolucida reconstruction of the morphology of a recorded dentate granule cell from a control rat (top) and voltage responses to intracellularly injected depolarizing and hyperpolarizing current pulses (500 ms duration, bottom). ***A2***, Top, *V*_m_ of the cell illustrated in ***A1*** during a 60 s recording. Bottom, Time–frequency power spectrogram (5 s sliding window, 0.2 s steps) corresponding to the top trace. Superimposed white line represents the time-varying power values in the SWO frequency range (0.1-2 Hz). Dashed white line (green arrow) indicates the SWO detection threshold used to detect SWO epochs (horizontal green bars). ***A3***, Top, Distribution of *V*_m_ values for the trace shown in ***A2***, Bottom, Relative proportion of DGCs according to the distribution of their *V*_m_ (Ske., skewed; Sym., symmetric; *n* = 10). ***B***, Same as in ***A*** for the post-SE condition. Note the presence of a continuous band in the slow-frequency range (∼0.8 Hz), and bimodal distribution of the *V*_m_ in the DGC from post-SE, but not control, rat (Bim., bimodal). ***C***, Power spectrum of the traces shown in ***A*** (blue) and ***B*** (red). ***D***, Average power spectrum for all recorded DGCs in control (blue line; *n* = 10) and post-SE (red line; *n* = 8). ***E***, Autocorrelogram of the traces shown in ***A*** (blue) and ***B*** (red). ***F***, Mean autocorrelogram for all recorded DGCs in control (blue line; *n* = 10) and post-SE (red line; *n* = 8). Light blue- and pink-shaded areas correspond to ±SEM.

**Figure 3.**
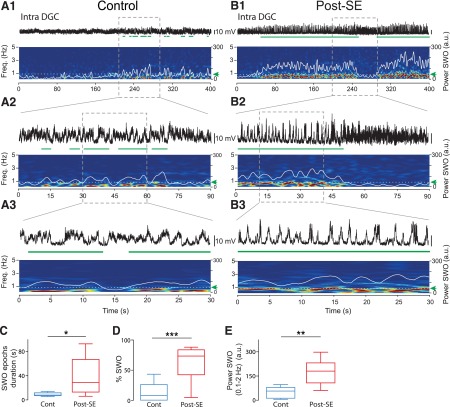
Increased slow-oscillatory epochs in the membrane potential of dentate granule cells in post-SE rats. ***A***, ***B***, Examples of detected SWO epochs in the membrane potential of dentate granule cells from a control rat (***A***) and a post-SE rat (***B***; spikes digitally removed). In each panel, the *V*_m_ (top), time–frequency power spectrogram (bottom) and time-varying power in the SWO band (0.1-2 Hz; superimposed white line) are represented at low (1) intermediate (2), and high (3) temporal resolution. In all cases, green horizontal bars below the trace highlight detected SWO epochs (Scale bars in ***B***, same as in ***A***). ***C***, Box plots of the duration of single SWO epochs detected in the *V*_m_ of DGCs in control (*n* = 6) and post-SE (*n* = 8) conditions. ***D***, Box plots of the percentage of recording time with significant oscillations in the SWO frequency range in the *V*_m_ of DGCs in control (*n* = 10) and post-SE (*n* = 8) rats. ***E***, Box plots of the power in the SWO frequency range averaged over all recorded cells and recording times in the *V*_m_ of DGCs in control (*n* = 10) and post-SE (*n* = 8) rats. For these and subsequent box plots, the box extends from the 25th to 75th percentile. The line in the middle of the box is the median. The whiskers go down to the smallest value and up to the largest. *p < 0.05; **p < 0.01; ***p < 0.001.

During slow-wave sleep and anesthesia, the *V*_m_ of neocortical cells rhythmically alternates between UP and DOWN states (Steriade et al., 1993, 2001; Cowan and Wilson, 1994). We first asked whether long-lasting epochs of UP/DOWN states could be detected in the *V*_m_ of DGCs from control and post-SE rats. We performed whole-cell patch-clamp recordings of DGCs *in vivo* in urethane-anesthetized control and post-SE rats (6–12 days after induction of a status epilepticus). Visual inspection of the *V*_m_ of DGCs from control rats did not reveal long-lasting epochs of UP/DOWN state modulation (Figs. 2*A*, 3*A*), as previously described (Isomura et al., 2006). In contrast, long-lasting epochs of UP/DOWN state modulation were observed in the *V*_m_ of the majority of DGCs from post-SE rats (Fig. 2*B*), interrupted by epochs of reduced slow oscillatory content (Fig. 3*B*). To automatically detect and compare epochs of slow oscillation in the *V*_m_ of DGCs, we developed a quantitative routine based on a single threshold set to the highest 90th percentile values of control DGCs’ *V*_m_ power in the SWO band (0.1-2 Hz; see also Sales-Carbonell et al., 2013; see Experimental procedure/Data analysis/Slow oscillatory epoch detection; Fig. 1*C*). Epochs of significant SWO power were detected in 6 of 10 DGCs from control rats. The duration of each SWO epoch was short (8.44 ± 1.26 s; range, 4.8-13.6 s; *n* = 6; Figs. 2*A*, 3*A*,*C*), and together they represented a small proportion of overall recording time (12.8 ± 4.90%; mean total duration of SWO per recording, 75 ± 28 s; mean recording time, 598 ± 25 s; *n* = 10; Fig. 3*D*). In contrast, epochs of strong SWO power could be detected in all DGCs from post-SE rats (*n* = 8 of 8). The duration of each SWO epoch was on average much longer than in the control condition (38.8 ± 11.3 s; range, 5.36–93.6 s; *n* = 8; Mann–Whitney rank sum test, *p* = 0.029^a^; Figs. 2*B*, 3*B*,*C*). Added up, these epochs represented a larger proportion of the total recording time (62.8 ± 10.2%; Student’s *t* test, *p* = 0.0006^b^; mean total duration of SWO per recording, 360 ± 66 s; mean recording time, 584 ± 49 s; *n* = 8; Fig. 3*D*). Over all recorded cells and recording time, the SWO band power of the Vm of DGCs was approximately three times higher in the post-SE versus the control condition (Mann–Whitney rank sum test, *p* = 0.004^c^; Fig. 3*E*). Power spectral analysis of the *V*_m_ of DGCs centered on these epochs revealed a high peak at ∼0.8 Hz in post-SE rats, while a smaller peak (Student’s *t* test, *p* = 0.004^d^) was observed at a lower frequency (∼0.4 Hz) in controls (Fig. 2*C*,*D*). Autocorrelograms of the *V*_m_ of DGCs during these epochs revealed a higher rhythmicity in the post-SE versus the control condition with larger negative peaks (minimum amplitude at trough, −0.26 vs −0.11; Mann–Whitney rank sum test, *p* = 0.037^e^; *n* = 8 and 10 cells, respectively; Fig. 2*E*,*F*) and second positive peaks in the post-SE condition, which were not present in controls. We conclude that the *V*_m_ of DGCs in post-SE rats strongly oscillates in the slow-frequency range for prolonged periods of time, unlike what is observed in controls.

We next wondered whether *V*_m_ dynamics were similar during SWO epochs in DGCs from control and post-SE rats. Analysis of the *V*_m_ distribution revealed in a majority of DGCs from post-SE rats a nonunimodal but bimodal distribution, a signature of strong UP/DOWN state dynamics (Hartigan’s dip test of unimodality, *p* = 0.017 ± 0.008; see Materials and Methods; Fig. 2*B3*; *n* = 5 of 8 cells). In the remaining cells, the *V*_m_ distribution was unimodal, either symmetrical (*n* = 1) or skewed (*n* = 2; see Materials and Methods). In DGCs from control rats, however, the distribution was always unimodal (Hartigan’s dip test of unimodality, *p* = 0.80 ± 0.25; *n* = 10; Isomura et al., 2006; Hahn et al., 2007), either skewed (*n* = 6) or symmetrical (*n* = 4; Fig. 2*A3*). These differences in *V*_m_ dynamics between DGCs from control and post-SE rats were not due to major differences in their intrinsic properties (Table 2).

**Table 2:** Dentate granule cells intrinsic properties

	Control (*n* = 10)	Post-SE (*n* = 8)	*p* value
Resting membrane potential (mV)	−79.1 ± 2.47	−74.8 ± 3.00	0.28^f^
Action potential threshold (mV)	−51.7 ± 1.10	−48.8 ± 0.48	0.08^g^
Action potential amplitude (mV)	55.9 ± 4.22	48.7 ± 5.44	0.30^h^
Action potential half-width (ms)	0.77 ± 0.03	0.80 ± 0.03	0.47^i^
Membrane input resistance (MΩ)	81.4 ± 8.27	94.9 ± 11.7	0.35^j^
Membrane time constant (ms)	11.2 ± 2.96	13.1 ± 1.88	0.06^k^

Values are represented as the mean ± SEM. All statistical comparisons were performed using the Student’s *t* test, except for Action potential threshold and Membrane time constant, where the Mann–Whitney rank sum test was used.

We conclude that SWO and UP/DOWN state behavior are major components of *V*_m_ dynamics in DGCs from post-SE rats, unlike what is observed in DGCs from control rats.

### Temporal relationship between the membrane potential of dentate granule cells and the neocortical slow oscillation in control and post-SE rats

**Figure 4.**
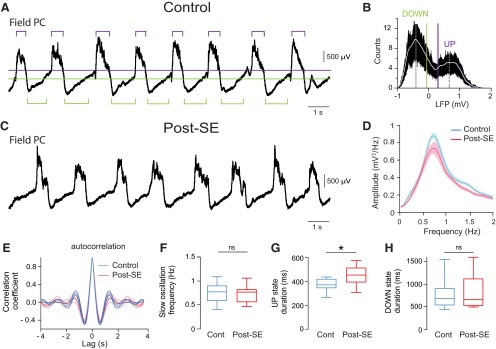
Comparing neocortical local field potential slow oscillations between control and post-SE rats. ***A***, Example LFP recorded in the parietal cortex in a control rat, with the threshold levels used to detect UP or DOWN states indicated by horizontal bars (green, DOWN state; purple, UP state). ***B***, Histogram of the distribution of LFP values for the trace shown in ***A***. The level for DOWN state detection (green vertical line) was set at the lower two-thirds of the distance between the peaks of the bimodal distribution of LFP values. The level for UP state detection (purple vertical line) was set at the higher two-thirds of the distance between the peaks of the bimodal distribution of LFP values. ***C***, Example LFP recorded in the parietal cortex from a post-SE rat. ***D***, Mean power spectrum of the LFPs recorded in the parietal cortex. Control (*n* = 10) and post-SE (*n* = 10) in ***D–H***. ***E***, Average autocorrelogram of LFPs in control (blue line) and post-SE (red line) conditions with a nonsignificant difference at the negative peaks. ***F***, Box plots of the frequency of the neocortical SWO in control and post-SE rats. ***G***, Box plots of neocortical UP state duration in control and post-SE rats. ***H***, Box plots of neocortical DOWN state duration in control and post-SE rats. Light blue- and pink-shaded areas in ***D*** and thinner lines in ***E*** indicate SEM. ns, *p* > 0.05; *p < 0.05. For description of box plots, see the legend of Figure 3.

Before investigating the temporal relationship between neocortical SWO and the *V*_m_ of DGCs, we first wondered whether neocortical SWO was modified during the latent period in TLE (Fig. 4). LFP recordings in the parietal cortex (which projects to the medial entorhinal cortex; Burwell and Amaral, 1998) revealed no change in SWO power (Student’s *t* test, *p* = 0.40^l^) and rhythmicity (Student’s *t* test, *p* = 0.71^m^) between control (*n* = 10) and post-SE (*n* = 10) rats (Fig. 4*A*,*C–F*). The only difference was a small but significant increase in UP phase duration (control rats, 374 ± 16 ms; post-SE rats, 450 ± 28 ms; *n* = 10; Student’s *t* test, *p* = 0.03^n^; Fig. 4*G*), while DOWN phase duration was not changed (Student’s *t* test, *p* = 0.74^o^; Fig. 4*H*). However, this difference did not result in a significant change of the parietal cortex SWO frequency (control rats, 0.75 ± 0.07 Hz; post-SE rats, 0.74 ± 0.06 Hz; *n* = 10; Student’s *t* test, *p* = 0.88^p^; Fig. 4*D*,*F*).

**Figure 5.**
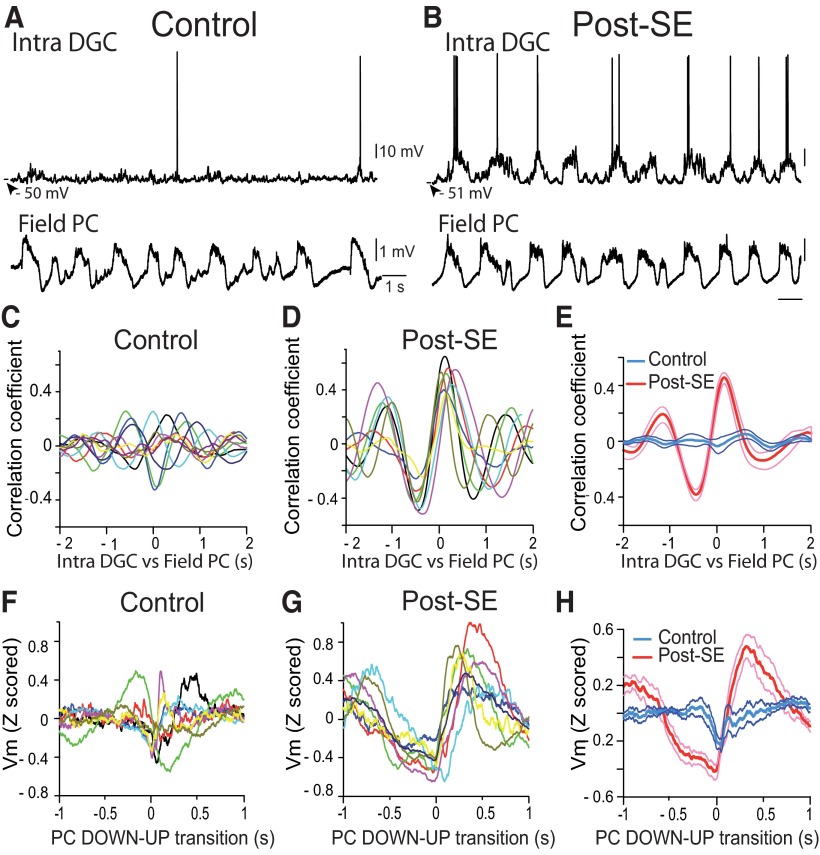
Strong temporal correlation between the membrane potential of dentate granule cells and the parietal cortex local field potential in post-SE rats, but not in control rats. ***A***, *V*_m_ of a DGC from a control rat (top) and simultaneously recorded LFP in the parietal cortex (bottom). ***B***, Same as in ***A*** for a DGC from a post-SE rat. Scale bars are as in ***A***. ***C***, *V*_m_ vs LFP cross-correlograms for all individual DCGs from control rats (*n* = 10). ***D***, Same as in ***C*** for DGCs from post-SE rats (*n* = 8). ***E***, Average *V*_m_ vs LFP cross-correlogram of all DGCs recorded in control rats (blue line, *n* = 10) and post-SE rats (red line, *n* = 8). ***F***, DOWN–UP transition-triggered *V*_m_ for all individual DGCs from control rats (*n* = 10). ***G***, Same as in ***F*** for DGCs from post-SE rats (*n* = 8). ***H***, Average DOWN–UP transition-triggered *V*_m_ for DGCs recorded in control rats (blue line, *n* = 10) and post-SE rats (red line, *n* = 8). Thinner lines in ***E*** and ***H*** represent ±SEM.

We next looked for a temporal coupling between the neocortical SWO in the parietal cortex and the *V*_m_ of DGCs. Cross-correlation between the *V*_m_ of DGCs and LFPs of parietal cortex was small and peaks were highly temporally jittered for DGCs recorded in control rats (*r* = 0.05 ± 0.03; one-sample Student’s *t* test, *p* = 0.15^q^; *n* = 10; Fig. 5*A*,*C*,*E*). In contrast, the correlation was high and highly significant in post-SE rats (*r* = 0.45 ± 0.04; one-sample Wilcoxon signed rank test, *p* = 0.008^r^; *n* = 8; Fig. 5*B*,*D*,*E*) with a fixed delay (166 ± 33.7 ms; *n* = 8). To better characterize the temporal relationship between activities recorded in the two structures we used the DOWN to UP transitions (DUT) of the neocortical SWO (see Materials and Methods) as a temporal reference to examine the *V*_m_ of DGCs (DUT-triggered *V*_m_; Fig. 5*F–H*; Isomura et al., 2006; Hahn et al., 2007). Examination of DUT-triggered averaged *V*_m_ values showed a positive bump at a fixed latency following neocortical DUT in all DGCs in post-SE rats (Fig. 5*G*). However, the individual responses in control rats were less consistent, often showing an absence of a clear bump after the neocortical DOWN–UP transition and in one case even a downward shift (Fig. 5*F*). In line with the results from individual cells, in post-SE rats, the mean DUT-triggered *V*_m_ average showed a clear bump with a peak at 317 ms following neocortical DUTs (Fig. 5*H*; *n* = 3543 DUTs in 8 cells), while no positive bump was observed in controls (Fig. 5*H*; *n* = 4864 DUTs in 10 cells; Student’s *t* test, *p* = 0.0005^s^ vs post-SE at peak).

We conclude that long-lasting UP/DOWN dynamics of the *V*_m_ of DGCs in post-SE rats is temporally locked to the neocortical SWO.

### Modulation of the firing of dentate granule cells by the neocortical slow oscillation in control and post-SE rats

**Figure 6.**
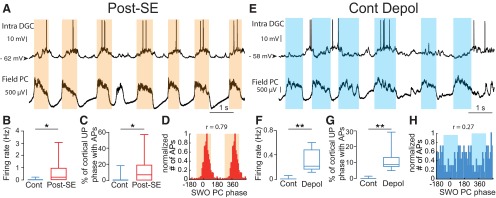
Modulation of the action potentials of dentate granule cells by the neocortical slow oscillation in post-SE and control rats. ***A***, ***E***, Membrane potential of a DGC (top) recorded in a post-SE rat (***A***) or a control rat after depolarization to induce spontaneous firing (***E***) and simultaneously recorded local field potentials in the parietal cortex (bottom). ***B***, Box plots of the firing frequency of intracellularly recorded DGCs from control rats (with no added depolarization; *n* = 10) and post-SE rats (*n* = 8). ***C***, Box plots of the percentage of neocortical UP phases associated with at least one AP in the control condition (with no added depolarization; *n* = 10) and post-SE condition (*n* = 8). ***D***, Phase distribution histogram of APs recorded in the DGC shown in ***A*** in reference to SWO phase in the parietal cortex. The orange-shaded area depicts the UP phase of SWO simultaneously recorded in the parietal cortex. ***F***, Same as in ***B*** for DGCs from control rats before and after depolarization to induce spontaneous firing (*n* = 8). ***G***, Same as in ***C*** for DGCs from control rats before and after depolarization to induce spontaneous firing (*n* = 8). ***H***, Same as in ***D*** for the cell illustrated in ***E***. *p < 0.05; **p < 0.01. For a description of box plots, see the legend of Figure 3.

We next aimed at determining whether the change in the dynamics of the *V*_m_ of DGCs observed in post-SE rats had any impact on their firing. We did this both at the single-cell level using intracellular recordings of the spiking activity of DGCs (Fig. 6) and at the network level using extracellular recordings of MUA in the dentate granule cell layer (see Fig. 8).

The *V*_m_ of DGCs from control rats was hyperpolarized (−79.1 ± 2.47 mV; *n* = 10), and most of them (80%) were silent (i.e. not firing any spontaneous action potential) during the recording time (mean recording time of silent cells, 892 ± 225 s; range, 410–2273 s; *n* = 8). The mean firing rate was low (active cells: 0.14 ± 0.08 Hz, *n* = 2; all cells: 0.03 ± 0.02 Hz, *n* = 10). Accordingly, few neocortical UP phases were associated with the firing of intracellularly recorded DGCs in controls (mean, 1.97 ± 1.81%; *n* = 10). In contrast, 75% of DGCs were spontaneously firing in post-SE rats (Fig. 6*A*,*B*; active cells: 0.84 ± 0.47 Hz, *n* = 6; all cells: 0.63 ± 0.37 Hz, *n* = 8; Mann–Whitney rank sum test, *p* = 0.029^t^ for all cells), and the number of neocortical UP phase associated with the firing of DGCs was significantly higher (Fig. 6*C*; mean = 13.4 ± 6.81%; Mann–Whitney rank sum test, *p* = 0.045^u^; *n* = 8). However, when spiking was observed during a neocortical UP phase, the mean number of spikes was not significantly higher in the post-SE versus the control condition (post-SE: 1.36 ± 0.16 spikes per neocortical UP phase, *n* = 524 UP phases with spikes in eight cells; control: 1.21 ± 0.06 spikes per neocortical UP phase, *n* = 86 UP phases with spikes in two cells; Mann–Whitney rank sum test, *p* = 0.64^v^). In post-SE DGCs, spikes were strongly and precisely modulated by the neocortical SWO (mean length of Rayleigh vector, 0.72 ± 0.06; mean phase dispersion, 41.2 ± 4.44 degrees; *n* = 6; Fig. 6*D*). We conclude that the firing of DGCs in post-SE rats is increased and highly modulated by the neocortical SWO.

**Figure 7.**
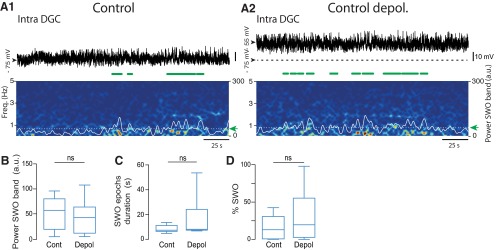
Lack of a significant increase in slow oscillations in the membrane potential of dentate granule cells by depolarization in control rats. ***A1***, ***A2***, Top, Membrane potential of a dentate granule cell from a control rat before (***A1***) and after (***A2***) artificial depolarization through direct current injection to ∼-55 mV (spikes digitally removed). Scale bar in ***A1*** is the same as in ***A2***. Bottom, Corresponding time–frequency power spectrograms (5 s sliding window, in 0.2 s steps). Superimposed white lines represent the time-varying power in the SWO band (0.1–2 Hz). Dashed white line (green arrow) indicates the SWO detection threshold used to detect SWO epochs highlighted by green horizontal bars below the traces. ***B***, Box plots of the mean power in the SWO band over all recorded cells (*n* = 8) and recording times before (Cont) and after (Depol) depolarization. *p* = 0.43^jj^; Paired Student’s *t* test. ***C***, Box plots of the mean duration of single intracellular SWO epochs before (Cont) and after (Depol) depolarization. *p* = 0.44^kk^; *n* = 6; Wilcoxon signed rank test. ***D***, Box plots of the percentage of intracellular SWO before (Cont) and after (Depol) depolarization. *p* = 0.24^ll^; *n* = 8; Paired Student’s *t* test. ns, *p* > 0.05. For a description of box plots, see the legend of Figure 3.

Because DGCs from control rats rarely fire or fire at a very low rate, it is difficult to determine whether their firing can be modulated by the neocortical SWO. To circumvent this problem, we artificially depolarized control cells to induce a firing rate comparable to that observed in DGCs from post-SE rats (mean Vm after depolarization, −56.5 ± 2.55 mV; *n* = 8 of 8 cells firing; mean firing rate, 0.29 ± 0.06 Hz; Mann–Whitney rank sum test, *p* = 0.02^w^ vs control rats; *p* = 0.72^x^ vs post-SE rats; *n* = 10 control DGCs; *n* = 8 control DGCs depolarized; *n* = 8 post-SE DGCs; Fig. 6*E*,*F*). Under depolarization, the number of neocortical UP phases associated with intracellular spiking was also significantly increased (paired Student’s *t* test, *p* = 0.004^y^ before vs after depolarization; Fig. 6*G*; Mann–Whitney rank sum test, *p* = 0.64^pp^ vs post-SE; *n* = 8). However, in this condition, spikes were significantly less strongly and less precisely modulated by the neocortical SWO than spikes from DGCs in post-SE rats (mean length of Rayleigh vector, 0.29 ± 0.07; Student’s *t* test, *p* = 0.0007^z^ vs post-SE; mean phase dispersion, 67.7 ± 3.47°; Student’s *t* test, *p* = 0.0005^aa^ vs post-SE; *n* = 8 control DGCs depolarized; *n* = 6 post-SE DGCs; compare Fig. 6*D*,*H*). Furthermore, the modulation of the *V*_m_ of control DGCs by the neocortical SWO was not significantly increased after depolarization (Fig. 7). Thus, when the spiking of control DGCs is artificially increased through direct depolarization, the modulation of their firing by the neocortical SWO is much weaker and less precise than that of DGCs from post-SE rats. Accordingly, their *V*_m_ does not show strong SWO modulation.

**Figure 8.**
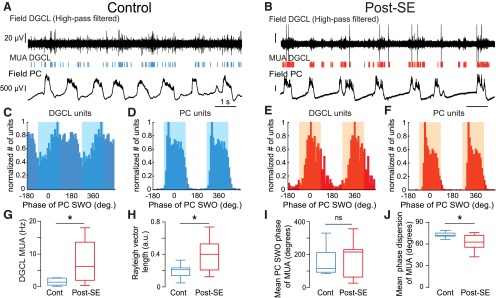
Increased rate and slow temporal modulation of multiunit activity in the dentate granule cell layer in post-SE vs control rats. ***A***, ***B***, High-pass-filtered (1000 Hz) local field potential recording in the dentate granule cell layer (DGCL; top) showing MUA (vertical bars) and simultaneously recorded local field potential in the parietal cortex (PC; bottom) in a control rat (***A***) and a post-SE rat (***B***). The scale bar values in ***B*** are the same as in ***A***. ***C***, ***D***, Phase histogram of MUA from the recording shown in ***A*** (***C***) and simultaneously recorded in the parietal cortex (***D***). The blue-shaded area depicts the UP phase of SWO in the parietal cortex. ***E***, ***F***, Same layout as ***C*** and ***D*** for the recordings illustrated in ***B***. The orange-shaded area depicts the UP phase of the SWO in the parietal cortex. ***G***, Box plots of the frequency of MUA recorded in the DGCL. *n* = 7 control and *n* = 17 post-SE rats in this and all subsequent panels. ***H***, Box plots of the length of Rayleigh vector of MUA recorded in the DGCL in control and post-SE rats. ***I***, Box plots of the preferred phase of MUA recorded in the DGCL for all recordings in control and post-SE rats in reference to SWO recorded in the PC. ***J***, Box plots of the dispersion of DGCL MUA around the mean phase of SWO recorded in the PC in control and post-SE rats. Ns, *p* > 0.05; *p < 0.05. For a description of box plots, see the legend of Figure 3.

**Figure 9.**
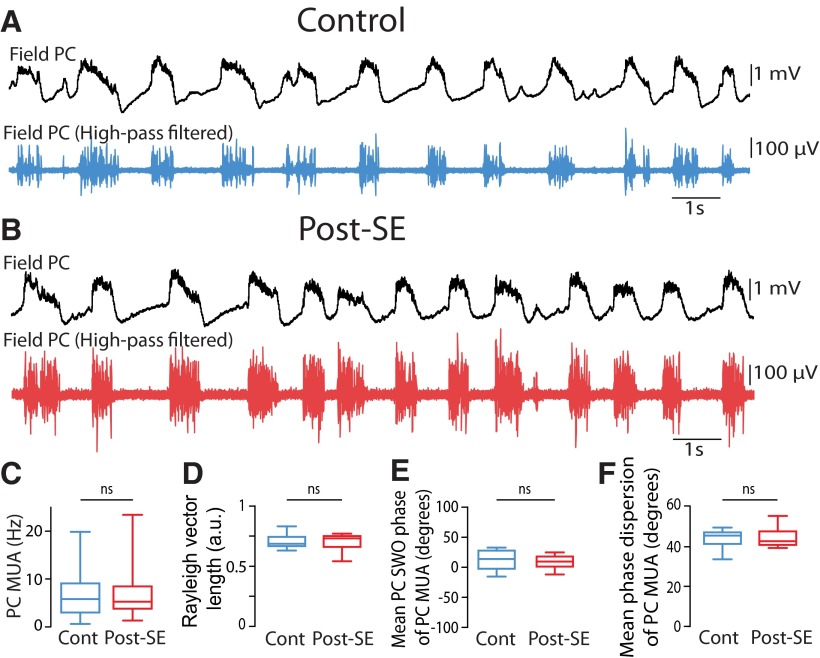
Modulation of neocortical multiunit activity by the slow neocortical oscillation in control and post-SE rats. ***A***, ***B***, Raw (top, black) and high-pass filtered (1000 Hz, bottom) local field potential showing MUA recorded in the parietal cortex from a control rat (***A***) and a post-SE rat (***B***). ***C***, Box plots of the frequency of MUA recorded in the parietal cortex. *n* = 14 control rats and *n* = 17 post-SE rats in this and all subsequent panels. ***D***, Box plots of the length of the Rayleigh vector in control and post-SE conditions. ***E***, Box plots of preferred SWO phase of PC MUA in control and post-SE conditions. ***F***, Box plots of the dispersion of PC MUA around the mean SWO phase for control and post-SE conditions. ns, *p* > 0.05. For a description of box plots, see the legend of Figure 3.

To see whether the increased spiking rate and SWO modulation of DGCs from post-SE rats could be observed at the network level, we next recorded MUA in the dentate granule cell layer of control and post-SE rats. MUA was significantly increased in post-SE (*n* = 17) versus control (*n* = 7) rats (Fig. 8*A*,*B*,*G*; 7.80 ± 1.44 Hz in post-SE rats; 1.61 ± 0.41 Hz in control rats; Mann–Whitney rank sum test, *p* = 0.01^bb^). We compared the strength of the modulation of MUA by the neocortical SWO in control and post-SE conditions (Fig. 8*C*,*E*). MUA in post-SE rats was more strongly modulated by the neocortical SWO (Fig. 8*H*; mean length of Rayleigh vector, 0.39 ± 0.04 in post-SE rats vs 0.19 ± 0.03 in control rats; Mann–Whitney rank sum test, *p* = 0.031^cc^), the mean phase at which MUA occurred was not different (Fig. 8*I*; Mann–Whitney rank sum test, *p* = 0.95^dd^), but the dispersion around the mean phase was significantly smaller in post-SE rats (Fig. 8*J*; mean phase dispersion, 61.6 ± 2.26° in post-SE rats vs 72.7 ± 1.47° in controls; Mann–Whitney rank sum test, *p* = 0.01^ee^). In sharp contrast, MUA recorded in the parietal cortex was not significantly increased in post-SE (*n* = 17) versus control (*n* = 14) rats (Mann–Whitney rank sum test, *p* = 0.86^ff^; Fig. 9*A–C*), nor was the strength of MUA modulation by the SWO (Student’s *t* test, *p* = 0.96^gg^; Figs. 8*D*,*F*, 9*D*), the mean phase (Student’s *t* test *p* = 0.48^hh^; Fig. 9*E*) or the phase dispersion around the mean (Student’s *t* test, *p* = 0.86^ii^; Fig. 9*F*).

## Discussion

In the present study, we investigated whether the impact of the neocortical SWO on DGCs, at the gate of the hippocampus, could be altered in an animal model of TLE. In line with this hypothesis, while the *V*_m_ of control DGCs weakly oscillated in the SWO frequency range for short periods of time, the *V*_m_ of most DGCs from post-SE rats showed strong UP/DOWN state dynamics for extended periods, time locked to neocortical SWO. Furthermore, the firing of DGCs was increased and strongly modulated by the neocortical SWO in post-SE rats, but not in control rats. Altogether, these results point to a major alteration in the processing of SWO, an important physiological pattern of activity, by DGCs during the early phase of TLE.

### Possible mechanisms of the switch to strong UP/DOWN state *V*_m_ dynamics in post-SE DGCs

A great deal of experimental and computational work has been conducted to understand the mechanisms leading to neocortical SWO and associated neuronal UP/DOWN state dynamics in the cortex (Timofeev et al., 2000; Bazhenov et al., 2002; Compte et al., 2003; Cossart et al., 2003; Shu et al., 2003; Haider et al., 2006; Holcman and Tsodyks, 2006; Rudolph et al., 2007; Chauvette et al., 2010). SWO has been proposed to result either from synaptic or intrinsic mechanisms. In the synaptic hypothesis, stochastic EPSPs could summate during the DOWN state and reach the activation threshold of intrinsic conductances, such as *I*_NaP_, initiating the UP state (Timofeev et al., 2000; Chauvette et al., 2010). Alternatively, SWO could result from the spontaneous activation of intrinsically bursting neurons in layer V of cortex (Le Bon-Jego and Yuste, 2007; Ló́rincz et al., 2015). Once an UP state is triggered, a barrage of synaptic excitatory and inhibitory inputs, occurring during the full duration of the UP state, contributes to its steady nature (Shu et al., 2003; Haider et al., 2006; Rudolph et al., 2007). This supposes a network with strong recurrent excitation (Sanchez-Vives and McCormick, 2000; Cossart et al., 2003) as well as feedback inhibition resulting from fast spiking basket cells activated during UP states (Shu et al., 2003; Fanselow and Connors, 2010; Massi et al., 2012; Tahvildari et al., 2012). The end of the UP state has been proposed to result from synaptic depression (Bazhenov et al., 2002; Holcman and Tsodyks, 2006), the buildup of activity-dependent potassium conductances (Sanchez-Vives and McCormick, 2000; Compte et al., 2003), and the activation of inhibitory interneurons (Shu et al., 2003) acting through GABA_A_ (Lemieux et al., 2015) and/or GABA_B_ (Parga and Abbott, 2007; Mann et al., 2009) receptors.

The lack of strong UP/DOWN state dynamics in control DGCs could result from the strong local inhibitory circuit of the dentate gyrus (Acsády et al., 1998; Nitz and McNaughton, 2004; Ewell and Jones, 2010; Sambandan et al., 2010) together with strong dendritic filtering (Soltesz et al., 1995; Schmidt-Hieber et al., 2007; Krueppel et al., 2011) that dampen the impact of excitatory inputs coming from layer II cells of the entorhinal cortex. In post-SE rats, however, the level of inhibition in DGCs is reduced during the latent period of TLE, notably at dendritic sites controlling inputs from the entorhinal cortex (Kobayashi and Buckmaster, 2003), presumably as a result of the early loss of several types of inhibitory interneurons in the dentate gyrus (Sloviter, 1987; Houser and Esclapez, 1996). In addition, excitatory inputs from layer II cells of the entorhinal cortex are increased 5 d post-SE as a result of decreased inhibition within the entorhinal cortex (Kobayashi et al., 2003). This imbalance in the excitatory/inhibitory drive could contribute to the strong UP/DOWN state *V*_m_ dynamics and firing observed in DGCs post-SE. Furthermore, at this early stage of epileptogenesis a switch of E(GABA) toward excitation has been reported (Pathak et al., 2007).

Alternatively, the absence of strong UP/DOWN state dynamics in control DGCs could result from the absence of a direct recurrent excitatory circuit in the dentate gyrus. Indeed, control DGCs only make indirect excitatory connections onto their peers through activation of mossy cells and need to fire at a sufficiently high rate to be able to discharge their targets (Henze et al., 2002). However, the axons of DGCs (the mossy fibers) sprout after status epilepticus to form a recurrent excitatory feedback circuit within the dentate gyrus not present in controls (Tauck and Nadler, 1985; Represa et al., 1987; Scharfman et al., 2003; Epsztein et al., 2005), which increases the excitability of DGCs (Artinian et al., 2011). These recurrent fibers result in part from the aberrant development of adult-born DGCs induced by the status epilepticus (Kron et al., 2010). This process is likely to be increased in our conditions because we use relatively young animals with a high level of adult neurogenesis and increased plasticity (Kuhn et al., 1996). Mossy fiber sprouting increases with time post-SE. It can already be observed during the latent period, but in a smaller proportion compared with the chronic stage (Wuarin and Dudek, 2001). Interestingly, computational work showed that even a low degree of mossy fiber sprouting can have a strong effect on network dynamics in a context of reduced inhibition (Santhakumar et al., 2005).

### Sleep slow oscillation, UP/DOWN states, and epilepsy

Epileptic discharges in focal epilepsy are frequently observed during non-rapid eye movement sleep (Malow et al., 1998). Most previous studies *in vivo* have focused on the impact of SWO on pathological activities, such as interictal epileptiform discharges or “UP spikes” (de Guzman et al., 2010; Bragin et al., 2012; Frauscher et al., 2015). No studies have analyzed the impact of the SWO itself on the *V*_m_ of principal cells in this pathological condition. Bragin et al. (2012) reported a decrease in the frequency of the field SWO recorded extracellularly in the dentate gyrus of chronically epileptic mice, due to an increased duration of both active and silent network phases. In our study, we tested the impact of the field neocortical SWO directly on the *V*_m_ of DGCs. We observed an increased duration and power of the *V*_m_ slow-oscillation of DGCs in post-SE versus control rats. Furthermore, the peak frequency of intracellular *V*_m_ slow oscillation was higher in the post-SE than the control condition (Fig. 2*D*). This frequency difference probably results from the fact that in controls, the *V*_m_ slow-oscillation of DGCs is poorly temporally coupled with the neocortical field activity (Fig. 5*E*). On the other hand, the *V*_m_ slow-oscillation of DGCs in post-SE rats is highly temporally coupled to the neocortical field SWO (Fig. 5*E*) and oscillates at the same frequency (compare Figs. 2*D*, 4*D*, red traces). Thus, the difference we observed between the *V*_m_ slow-oscillation frequencies likely reflects an increased temporal coupling in the post-SE condition rather than an absolute change in the frequency of the neocortical field SWO, which is not different (Fig. 4*D*).

The only study on the consequences of a status epilepticus on intracellular UP/DOWN state *V*_m_ dynamics was performed *in vitro* using neocortical slices (Gerkin et al., 2010). In that work, the frequency of UP states recorded in layer 2/3 neocortical pyramidal neurons was increased 24 h after a picrotoxin-induced seizure. This resulted from shorter DOWN states, associated with increased single-cell intrinsic excitability. Indeed, a higher number of action potentials was generated per UP state following seizure induction. However, in our study, the frequency of the SWO was not modified in the parietal cortex, and neocortical MUA activity was not increased, suggesting no change of neocortical excitability during the latent period in our model of TLE. In contrast to neocortical neurons in controls, which display strong UP/DOWN state dynamics, the Vm of control DGCs was only weakly modulated by the neocortical SWO and clear UP/DOWN dynamics were rarely observed. However, several days after the induction of the status epilepticus, clear UP/DOWN dynamics could be seen. The scarcity of UP/DOWN *V*_m_ dynamics in controls and the abundance following the SE points to a morphofunctional change in the network, rather than a change in the intrinsic excitability of DGCs, as a possible explanation. In line with this hypothesis, increasing single-cell excitability through direct current injection via the recording pipette was not enough to induce SWO in the *V*_m_ of control DGCs. Furthermore, the number of spikes per neocortical UP phase was not modified in DGCs from post-SE rats, arguing against an increased intrinsic excitability.

### Functional consequences of higher UP/DOWN state *V*_m_ dynamics in DGCs from post-SE rats

At the entrance of the hippocampus, the dentate gyrus has been proposed to transform dense neocortical inputs into sparse and specific neuronal representations (Chawla et al., 2005; Acsády and Kali, 2007; Leutgeb et al., 2007), which can then be imposed onto their downstream CA3 pyramidal cells target for subsequent storage (McNaughton and Morris, 1987; Treves and Rolls, 1992). In accordance with this function, DGCs are particularly reluctant to fire action potentials both *in vitro* (Scharfman, 1991; Ewell and Jones, 2010) and *in vivo* (Jung and McNaughton, 1993; Penttonen et al., 1997; Leutgeb et al., 2007; Neunuebel and Knierim, 2012; Pernía-Andrade and Jonas, 2014; Diamantaki et al., 2016; Kowalski et al., 2016). By bringing DGCs closer to firing threshold, UP states likely facilitated the generation of action potentials. Indeed, 75% of DGCs were spontaneously firing in post-SE rats against 20% in controls, and the firing rate of individual cells was strongly increased. This increase likely results from the increased number of UP states rather than increased intrinsic excitability of DGCs, because the number of spikes generated by individual UP states was not different between control and post-SE rats. The MUA recorded in the dentate granule cell layer was not only increased, but also was more strongly and precisely paced by the neocortical SWO in post-SE versus control rats. Previous studies (Mori et al., 2004) have shown that DGCs can have either an inhibitory (at low firing rate) or an excitatory (at high firing rate) impact on downstream targets. In our recordings, the increase in the firing rate of individual cells, although substantial, was still too low to allow faithful excitation of CA3 pyramidal cells during the UP state by individual DGCs (Henze et al., 2002). The increased frequency could instead enhance their inhibitory impact on CA3 activity during the neocortical UP state already observed in controls (Isomura et al., 2006). On the other hand, because more DGCs are firing spontaneously and because their firing is more strongly synchronized by the SWO, the likelihood of having an ensemble of DGCs coactive will be increased. Altogether, these cells could have a significant impact on single-CA3 pyramidal cell firing behavior. The net impact of these changes on downstream targets remains to be investigated but is difficult to anticipate because CA3 and CA1 networks are themselves profoundly modified in TLE.

The slow cortical oscillation is able to bias the occurrence of hippocampal sharp-wave/ripple events (Sirota et al., 2003; Battaglia et al., 2004; Mölle et al., 2006; Logothetis et al., 2012) and is important for hippocampus-dependent declarative memory consolidation in humans (Marshall et al., 2006). Future studies will tell whether the increased modulation of the activity of DGCs by the slow cortical oscillation that we report has an incidence on the replay and consolidation of information during sleep in patients with TLE.

## Synthesis

The decision was a result of the Reviewing Editor Menno Witter and the peer reviewers coming together and discussing their recommendations until a consensus was reached. A fact-based synthesis statement explaining their decision and outlining what is needed to prepare a revision is listed below. The following reviewer(s) agreed to reveal their identity: Laura

In this study the authors examine the relation between neocortical slow-wave oscillations and the activity of dentate granule cells in the latent phase of temporal lobe epilepsy in anesthetized rats. Slow-wave oscillations (SWO) has been shown to be important for memory formation and the communication between neocortex and hippocampus. The dentate gyrus is suggested to function as a gate between incoming neocortical information and the hippocampus. The current study combines local field recordings of SWO in neocortex with intracellular recordings of DGCs membrane potential in vivo and reveals an abnormally strong influence of neocortical SWO on DGCs membrane potential, providing new knowledge about hippocampo-neocortical interaction during the early phase of TLE.To the best of my knowledge this is the first study on the consequenses of a status epilepticus on intracellular UP/DOWN state membrane potential dynamics of dentate granule cells in vivo and thus advances the field by showing that slow wave oscillations become abnormally synchronized between the neocortex and the dentate gyrus following an epileptic seizure.

Although the visual abstract captures the main findings (maybe the power-spectra are unnecessary), there is one mistake that needs to be corrected: the visual abstract implies that parietal cortex projects to dentate gyrus, but this is incorrect and does not match what was reported in the anatomy paper (Burwell and Amaral, 1998). A similar incorrect statement is made in the text and should be corrected. Burwell and Amaral, 1998 report that parietal cortex projects to medial entorhinal cortex. Whether or not that information is passed on to the dentate gyrus might be a logical suggestion, but it has not been shown yet.

The paper is of interest, well written, and the results appear to be convincing. However, there are several results and methods in the manuscript that are not clearly explained. The reviewers have raised the following comments:

Major points:

1.Why are different measures used in the group data bar plots in Figures 1 and 3? Intracellular recordings vs. LFP? Dentate gyrus vs. parietal cortex? This should be explained. The authors claim that differences are seen between controls and post-SE animals in dentate gyrus but not in parietal cortex, but different measures were used in the different brain regions.

2.Related to the first point, it is unclear why SWO phase is shown in Figure 7C-F, whereas up-down state phase is shown in Figure 8C-D. What is the difference? Why were different measures employed to measure the phase of the parietal oscillation in these two figures? The results section is written as though the same measure was used in Figure 8C&amp;D as in Figure 7D&amp;F. If so, then the data in Figure 8C&amp;D would be exactly the same data that was shown in Figure 7D&amp;F.

3.The examples in Figure 6A1 &amp; A2 are not representative of the group data and are potentially misleading. The bar plots show roughly equal power in the SWO band between control and post-depolarization recordings, non-significantly longer duration SWO events in the post-depolarization recordings compared to controls, and a non-significantly higher percentage of SWO events in post-depolarization recordings compared to controls. However, the examples make it appear as though fewer SWO events occur with lower power and for a shorter duration in post-depolarization recordings compared to control recordings. These examples thus imply that depolarizing current injection subsequently reduces SWO in control rats, but that is not what is shown in the group data in Figure 6B-D.

4.On page 10, lines 24-25, the method in the following passage was not described clearly and does not match the method as shown and describe in Figure 2C and the Figure 2C legend: "The average of thresholds calculated for each control DGC was used as a unique threshold for SWO periods detection in control and post-SE DGCs." This text implies that each control DGC had its own unique threshold, but Figure 2C (and the Figure 2C legend) state that a single threshold was defined as the mean across all control cells.

5.On page 11, line 20, the following method is not described in sufficient detail and does not seem to match the method as it is described in Figure 3B: "Briefly, the thresholds for the detection of different states were set at equal distances between the peaks of the bimodal distribution of neocortical LFP values". This makes it sound as though the thresholds were set as the midpoint between the two peaks in the bimodal distribution, but this is not what is shown in Figure 3B and does not match how the method is described in the Figure 3B legend.

6.On lines 20-22 on page 10, I felt that the method in the following passage was not described clearly: "The total power of Vm in the SWO band was computed using a 5 s sliding window advancing in steps of 0.2 s (Fig. 2A and B, bottom) and used to perform a distribution (Fig. 2C)." What exactly does "used to perform a distribution" mean?

7.On page 15, lines 13-15, the authors write, "To better quantify the  amount of Vm oscillation in the slow frequency range (SWO: 0.1 to 2 Hz) we adapted a  quantitative routine (Sales-Carbonell et al., 2013; see experimental procedure; Fig. 2)." It is unclear to which quantitative routine the authors are referring. Which part of Fig. 2? Which part of experimental procedures (i.e., Materials and Methods)?  

8.In Figure 2E1, post-SE, what happens at the ∼260 ms time point when all SWO power is essentially lost?

9.In Figure 7A&amp;B, the authors should clarify in the figure legend that the vertical lines depict multiunit activity in DG (if that is indeed the case) since panels C&amp;D subsequently show results from both DG and parietal cortex units.

Minor points:

1.The first two sentences of the Results do not follow logically from one to the next. The first sentence discusses the membrane potential of neocortical cells, but then the second sentence states, "We first looked for differences in Vm dynamics between" dentate gyrus cells from control and post-epileptic rats. The transition from neocortical cells to dentate gyrus cells is abrupt and confusing.

2.Page 4: The authors state that Vm of hippocampal neurons are not affected by neocortical UPS. This is of course correct when it comes to principal neurons, but it has been shown that R-LM interneurons are phase locked to neocortical UDS (Hanhn et al 2006).

3.On page 23, lines 24-25, the authors write, "Epileptic discharges in focal epilepsy are frequently observed during non-rapid eye movement sleep", but no reference is provided to back up this statement.

4.On page 28, line 18, "periods were the time varying SWO power is above SWOThr" should be "periods where the time varying SWO power is above SWOThr".

On page 32, line 15, "MUA recorded DGC layer" should be "MUA recorded in the DGC layer".

5.Quite a few non-standard abbreviations are used throughout the text (e.g., "SWOThr"). Are all of these abbreviations necessary? They affect the paper's readability.

6.The fact that these results points to a change in the network rather than a change in DGCs intrinsic excitability might be worth including in the abstract.

7.Animals in the current study are young (P27-P43) and some of them within the age of typical "critical period" (P25-30) with hightened plasticity. I would like the authors to briefly discuss any influence of age on the results.

8.Is Fig. 2E described/cited in the main text? (I could not find mention of it.) If not, perhaps it should be.

9.Many of the bar charts in the manuscripts could be replaced by more appropiate and informative box plots (see Nature Methods 11, 113 (2014) doi:10.1038/nmeth.2837).

10.Figure 2 explain the method behind the results shown in figure 1CD. I recommend either moving figure 1CDE to figure 2 or moving figure 2 to experimental procedures.

11.Figure 1 A &amp; B is a bit overcrowded. I think it would be better if A3 and B3 were moved. It would perhaps be better for the reader (and more striking) if the two skweness-bimodality charts were on the same chart.

12.Figure 5: I assume the most interesting comparison in this figure is between post-SE and the depolarized control, and the control bars in B&F represent the same data. Thus, wouldn't it be better to merge B&F and C&G ?

13.Figure 8: There seems to be a shift in the phase of PC units vs UDS phase post-SE. In control condition most units fire at the start of the upstate and post-SE most units fire at the end. This is not addressed with the current quantative analysis/statistics.
